# Hawthorn fruit acid consumption attenuates hyperlipidemia-associated oxidative damage in rats

**DOI:** 10.3389/fnut.2022.936229

**Published:** 2022-08-03

**Authors:** Yicheng Feng, Shan Gao, Ting Zhu, Guibo Sun, Peisen Zhang, Yichun Huang, Shuang Qu, Xiaomeng Du, Dehua Mou

**Affiliations:** ^1^Beijing Advanced Innovation Center for Soft Matter Science and Engineering, College of Life Science and Technology, Beijing University of Chemical Technology, Beijing, China; ^2^College of Food and Biology, Hebei University of Science and Technology, Shijiazhuang, China; ^3^Institute of Medicinal Plant Development, Chinese Academy of Medical Sciences and Peking Union Medical College, Beijing, China; ^4^Beijing Obstetrics and Gynecology Hospital, Capital Medical University, Beijing Maternal and Child Health Care Hospital, Beijing, China

**Keywords:** hawthorn fruit acid, hyperlipidemia, cholesterol, anti-oxidation, atherosclerosis

## Abstract

**Context:**

Hyperlipidemia is a highly prevalent risk factor for atherosclerosis and stroke. The currently available medications used to treat Hyperlipidemia cannot improve its oxidative stress damage. Consumption of hawthorn can regulate blood sugar and blood lipids, and its rich fruit acid is a natural antioxidant that can improve oxidative stress damage.

**Objective:**

The present research aimed to investigate the protective effect of hawthorn fruit acid (HFA) on hyperlipidemia and to determine its potential molecular mechanism.

**Materials and methods:**

Sprague-Dawley rats were fed a high-fat diet (HFD) to induce hyperlipidemia and treated orally with hawthorn fruit acids (HFA). Serum and liver levels of total cholesterol (TC), triglycerides (TG), high-density lipoprotein cholesterol (HDL-C), superoxide dismutase (SOD), hydrogen peroxide (CAT), and malondialdehyde (MDA) were measured. Human hepatocellular carcinoma cell lines (HepG2) cells were treated with 0.1 mM oleic acid and HFA (0.125, 0.25 mg/mL), and intracellular TC, TG, HDL-C, SOD, CAT and MDA were measured. Changes in LDLR, HMGCR, Nrf2, HO-1, NQO1 protein and gene expression were analyzed by Western blot and qPCR.

**Results:**

This study found that HFA treatment effectively reduced the level of triglyceride, cholesterol, and glucose, and attenuated hepatic steatosis in rats. Additionally, oxidative stress damage of rats was effectively reduced by treatment with HFA. Western blot and qPCR analysis indicated that HFA treatment inhibited fat accumulation in HepG2 cells by upregulating LDLR and downregulating HMGCR gene expression. HFA inhibits oleic acid (OA)-induced oxidative damage to HepG2 by activating the Nrf2/HO-1 signaling pathway.

**Conclusion:**

HFA administration can provide health benefits by counteracting the effects of hyperlipidemia caused by an HFD in the body, and the underlying mechanism of this event is closely related to the activation of the Nrf2/HO-1 signaling pathway.

## Introduction

Hyperlipidemia is a metabolic disorder in which blood lipid levels are abnormally elevated (1–3). Due to exceptionally high lipid levels, excess lipids in the blood can accumulate in the walls of the arteries, thus inducing chronic diseases such as cardiovascular disease, stroke, and atherosclerosis (4, 5). An unbalanced diet is a cause of hyperlipidemia (3). High-fat diets (HFD) lead to oxidative stress damage by increasing reactive oxygen species (ROS) and decreasing antioxidant enzymes (6, 7). Prolonged exposure of cells to ROS leads to an imbalance in adipocytokine expression and impaired lipid metabolism (8, 9). Increased ROS production from accumulated fat leads to increased oxidative stress in the blood, which may severely affect other organs (9). Therefore, inhibition of oxidative stress in hyperlipidemic states is considered an essential therapeutic approach (10). Current anti-hyperlipidemic drugs include mainly statins, fibrates, and bile acid chelators; however, these drugs are not effective in reducing oxidative stress (6, 11, 12). In recent years, the use of synthetic antioxidants has been limited due to their possible toxicity and carcinogenicity, and natural antioxidant products have received increasing attention (13, 14).

Oral consumption of hawthorn can lower blood lipid levels, regulate blood glucose and antioxidants, etc (15–17). Hawthorn has recently become an influential component of nutrition and nutraceuticals due to its beneficial effects on the prevention of cardiovascular disease (18). More than 150 substances have been identified and isolated from hawthorn, including a large number of fruit acids, such as gallic acid, chlorogenic acid, ferulic acid, and P-coumaric acid (16, 18). Fruit acids that enable nutrient digestion and stimulate blood circulation are one of the quality indicators of hawthorn fruit (18). Many data suggest that the administration of fruit acids has strong antioxidant activity and hypolipidemic effects (8, 19–22). However, studies on the hypolipidemic and antioxidant mechanisms of hawthorn fruit acids are not clear. Therefore, we used an oleic acid-induced HepG2 cell model and an HFD-induced rat model to evaluate the therapeutic potential and mechanism of hawthorn fruit acids on hyperlipidemia.

## Materials and methods

The hawthorn variety is *Crataegus pinnatifida Bunge* cv. “Waibahong,” taken from Xinglong County, Chengde City, Hebei Province. The fresh hawthorn was freeze-dried in a vacuum freeze dryer (VaCo 2, Germany ZIRBUS Co., Ltd.), crushed with a crusher, and passed through a 100-mesh sieve to obtain hawthorn powder for later use.

Oxalic acid (OxA, C_2_H_2_O_4_, CID:971), Tartaric acid (TA, C_4_H_6_O_6_, CID:875), L-malic acid (L-MA, C_4_H_6_O_5_, CID:222656), Ascorbic acid (AA, C_6_H_8_O_6_, CID:54670067), Citric acid (CiA, C_6_H_8_O_7_, CID:311), Gallic acid (GA, C_7_H_6_O_5_, CID:370), Chlorogenic acid (ChA, C_16_H_18_O_9_, CID: 1794427), Caffeic acid (CaA, C_8_H_9_O_4_, CID:689043), P-coumaric acid (P-CA, C_9_H_8_O_3_, CID:637542), Ferulic acid (FA, C_10_H_10_O_4_, CID:445858) are all bought from Aladdin (Shanghai, China). Simvastatin (C_25_H_38_O_5_, CID:54454) was purchased from Yuanye Bio (Shanghai, Guangzhou). β-actin (AC038), Nrf2 (A0674), HO-1 (A1346), NQO1 (A19586), HMGCR (A19063), LDLR (A14996) were purchased from ABclonal (Wuhan, China).

### Hydroalcoholic extraction of hawthorn fruit

A certain amount of hawthorn powder was extracted with microwave assistance at 50% ethanol concentration, 28:1 (mL/g), 34 min, and 75°C. The supernatant was obtained by centrifugation and passed through 0.22 μm filter membrane for measurement.

### Determination of hawthorn fruit organic and phenolic acids composition

Hawthorn organic acids and phenolic acids were separated using a high-performance liquid chromatograph (LC-20A) from Shimadzu, Japan. Organic acids were quantified on a Dima Platisil ODS C18 [4.6 × 250 mm ID, 5 μm] column. The mobile phase was a 0.02 mol/L NaH_2_PO_4_ (pH 2.9): acetonitrile (98: 2) buffer solution. The sample (20 μL) was separated on the column at 37°C with a mobile phase flow rate of 0.5 mL/min; the absorbance was monitored at 213 nm with a UV detector. The peaks of organic acids were identified according to their retention times and quantified using an external standard curve. Phenolic acids were quantified on a Dima Platisil ODS C18 [4.6 × 250 mm ID, 5 μm] column. The mobile phases were solvent A (methanol: acetic acid: water = 10:2:88) and solvent B (methanol: acetic acid: water = 90:2:8). The samples (20 μL) were separated on a column at 37°C with a gradient elution program of 0% to 15% for B from 0 to 25 min, 15 to 50% for B from 25 to 45 min, and 50 to 0% for B from 45 to 53 min. The mobile phase flow rate was 1 mL/min; the absorbance was monitored at 280 nm with a UV detector. The peaks of phenolic acids were identified according to their retention times and quantified using an external standard curve.

### *In vitro* lipid peroxidation analysis

A healthy male SD rat was sacrificed after fasting for 12 h and then the blood samples were taken in a heparin tube. Saline was added at 0–4°C, the supernatant was discarded after centrifugation, and the supernatant was repeatedly washed and discarded to prepare 0.5% erythrocyte suspension. Take 0.5 mL of erythrocyte suspension and add 1 mL of saline. Then add 0.1 mL of different concentrations of Hawthorn extract, 0.1 mL of deionized water for the blank control group, and 0.1 mL of Vitamin C (VC) corresponding to the concentration of Hawthorn extract for the positive control group, respectively. After mixing in a constant temperature water bath at 37°C for 60 min, 0.3 mL of 20% trichloroacetic acid was added in a water-cooled shake, and 2 mL of 0.1 mol/L hydrochloric acids were added. After centrifugation, 3 mL of the supernatant on the supernatant was added with 0.67% thiobarbituric acid 1 mL, boiling water bath for 15 min, and absorbance was measured at 532 nm after cooling. The obtained lipid oxidation inhibition rate was shown in equation (1).


(1) $$\text{RBC lipid eroxidation inhibition rate \% = }\frac{\text{A0}-\text{A1}}{\text{A0}}\times 100\%$$

A healthy male SD rat was sacrificed after fasting for 12 h and then the blood samples were taken in a heparin tube. The livers were removed and ground into 5% liver homogenate in saline at 0–4°C. Take 1mL of liver homogenate, then add 0.1 mL of Hawthorn extract at different concentrations, 0.1 mL of deionized water for the blank control group, and 0.1 mL of VC corresponding to the concentration of Hawthorn extract for the positive control group. After mixing in a constant temperature water bath at 37°C for 60 min, 1 mL of 20% trichloroacetic acid and 1 mL of 0.67% thiobarbituric acid were added after water-cooled shaking. After cooling in a boiling water bath for 15 min, the centrifuged clear solution was taken at 532 nm absorbance. The obtained lipid oxidation inhibition rate was shown in equation (2).


(2) $$\text{Inhibition rate of liver lipid eroxidation \% = }\frac{\text{A0}-\text{A1}}{\text{A0}}\times 100\%$$

### Cytotoxicity

HepG2 cells were obtained from the Institute of Medicinal Plants, Peking Union Medical College. The HepG2 cell culture method was reported in the literature (23). The cell viability assay was reported in the literature (24). 0, 0.125, 0.25, 0.5, 1, 2 mg/mL HFA and 0, 0.05, 0.1, 0.2, 0.25, 0.3 mM OA treatments were applied.

### Cell culture and hawthorn fruit acid treatment

After the cells grew to 80% confluence, the medium was changed and drugs were added. The experiments were divided into five groups. The groups were as follows: (i) control group; (ii) OA treated group; 0.1 mM OA incubated for 18 h; (iii) OA + simvastatin group; 100 μM OA and 5 μM simvastatin incubated for 18 h; (iv) OA + HFA high dose group; 100 μM OA and 0.25 mg/mL HFA incubated for 18 h; and (v) OA + HFA low dose group; 100 μM OA and 0.125 mg/mL HFA incubated for 18 h.

### Oil red O staining

Stain lipids in HepG2 cells using Oil Red O solution. Observe the stained cells under a microscope (EVOSFL Color) and capture the images.

### Cell biochemical index analysis

The intracellular accumulation of TC, TG, HDL-C, LDL-C, MDA, CAT, GSH-Px, GSH, and SOD was determined according to commercial kits (Nanjing Jiancheng Bioengineering Institute, Nanjing, Jiangsu, China). Operation was conducted according to the commercial kit instructions.

### Quantitative real-time polymerase chain reaction

The RT-qPCR method was reported in the literature (25). RT-qPCR was performed on the LightCycler 96 thermal cycler using a fluorescent PCR kit (SYBR Premix Ex TaqTM, Takara, United States). The primer sequences are shown in Supplementary Table 1.

### Western blot analysis

Proteins were extracted using RIPA lysis buffer containing phosphatase and protease inhibitors (26). Aliquots containing the same number of proteins were separated on a 10% sodium dodecyl sulfate-polyacrylamide gel and then transferred to a PVC membrane for immunoblotting. β-actin, Nrf2, HO-1, NQO1, HMGCR, and LDLR were all diluted 1000 times for use. A semi-quantitative analysis of the ratio of each target protein to the grayscale value of the internal β-actin reference was performed.

### Animal experiments

Male rats were more sensitive to high-fat feeding and had more pronounced insulin resistance and decreased glucose tolerance than females. Therefore, we selected male rats to establish a hyperlipidemia model. Male Sprague-Dawley (SD) rats, weighing 230–270 g, from Hebei Medical University (Shijiazhuang, China), housed in an animal housing room at a constant temperature (25 ± 1°C) for 12 h with fixed darkness and light cycles (Permit No. SCXK2018-004, Laboratory Animal Quality Certificate No. 1804016).

In total of 48 male rats were randomly divided into the following 6 groups. Control group: normal diet + 0.9% saline solution treatment; model group: HFD + 0.9% saline solution treatment; simvastatin group: HFD + 1.8 mg/kg⋅d^–1^ simvastatin; high-dose group: HFD + 100 mg/kg⋅d^–1^ HFA extract; low-dose group: HFD + 30 mg/kg⋅d^–1^ HFA extract; Compound group: HFD + 100 mg/kg⋅d^–1^ HFA composite (HFA composites prepared from oxalic acid, tartaric acid, L-malic acid, ascorbic acid, citric acid, gallic acid, chlorogenic acid, caffeic acid, p-coumaric acid, and ferulic acid standards based on the results of HPLC assay). Except for the control group, the other 5 groups were fed with HFD for 2 weeks, and then administration started. These drugs were taken for 8 weeks, with each animal weighed every week, and blood collected from the tip of the tail. See Supplementary Table 2 for dietary information.

### Biochemical parameters

The cumulative amount of serum and liver homogenate of TC, TG, HDL-C, LDL-C, MDA, CAT, SOD, GSH-px, and T-AOC was measured using commercial kits. Operation was conducted according to the commercial kit instructions. The atherosclerosis index (AI) was calculated based on the content of T-CHO and HDL-C in the serum.


(3) AI=(TC-HDL)÷HDL

### Pathology

The liver tissue was cut into 4-μm slices and stained with hematoxylin and eosin stain. All sections were examined by light microscopy (Nikon Primo Star microscope, Carl Zeiss, Germany).

### Statistical analysis

All data are expressed as the means ± standard deviation (SD) and analyzed by GraphPad Prism 8.0. One-way ANOVA was used to assess differences between multiple groups. Statistically significant results were indicated as*p < 0.05, ^**^p < 0.01, ^***^p < 0.001, and ^****^p < 0.0001 vs. control group; ^#^*p* < 0.05,^##^*p* < 0.01, ^###^*p* < 0.001, and ^####^*p* < 0.0001 vs. model group.

## Results

### Hawthorn fruit organic and phenolic acids composition

**FIGURE 1:**
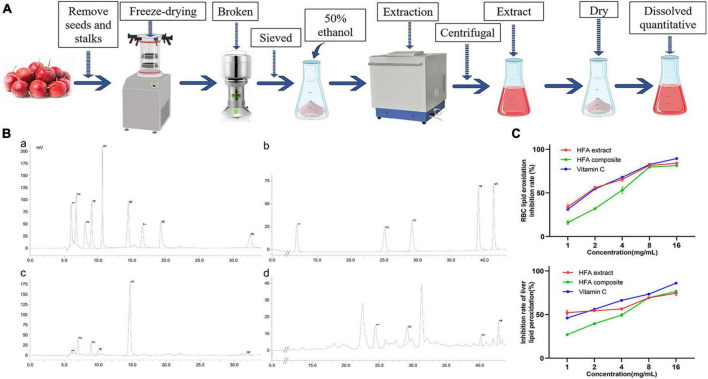
Identification of hawthorn fruit acids (HFA) and the *in vitro* antioxidant capacity of HFA. **(A)** Flow chart of HFA preparation; **(B)** high-performance liquid chromatogram; a. Peaks 1–9 are OxA, TA, PA, L-MA, AA, CiA, SA, D-MA, GA; b. Peaks 1–5 are GA, PA, ChA, CaA, P-CA, FA; c. Peaks 1–6 are OxA, TA, L-MA, AA, CiA, GA; d. Peaks 1–4 are ChA, CaA, P-CA, FA; **(C)** *in vitro* lipid peroxidation analysis. Data express mean ± SD (*n* = 3).

Figure 1A is a flow chart of the extraction of organic and phenolic acids from hawthorn. Based on the quantification of the standard curve of fruit acids determined by HPLC (Supplementary Figure 1), we determined that hawthorn extract contained 10 acids, namely OxA, TA, L-MA, AA, CiA, GA, ChA, CaA, P-CA, and FA (Figure 1B). Among them, citric acid had the highest content of 147.67 mg/g. The main components of phenolic acids were chlorogenic acid and ferulic acid with 1.04 mg/g and 0.84 mg/g, respectively (Supplementary Table 3).

The antioxidant capacity of HFA was initially verified by measuring its anti-lipid peroxidation level *in vitro*. Figure 1C shows that HFA administration inhibited lipid peroxidation in both liver tissue and erythrocytes. HFA treatment has a more stable anti-lipid peroxidation ability than VC and is a good antioxidant.

### The lipid-lowering effect of hawthorn fruit acid on the HepG2 cells

**FIGURE 2:**
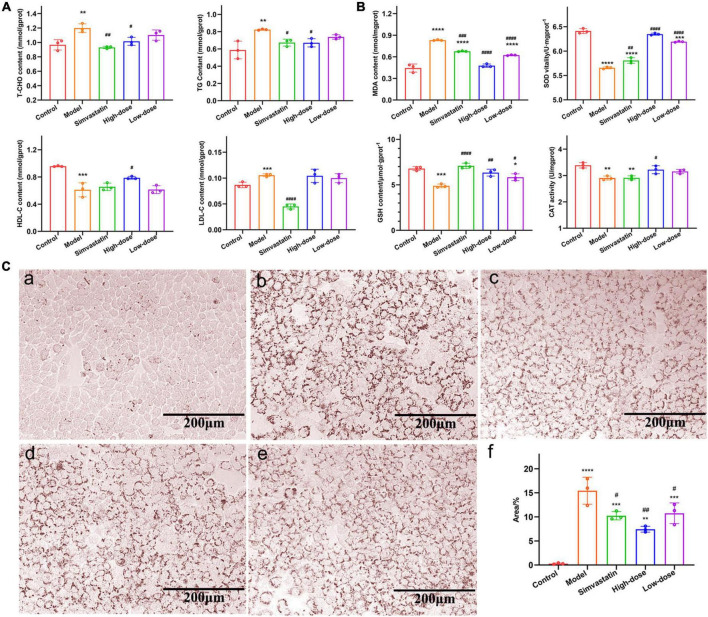
Effect of hawthorn fruit acid (HFA) on lipid accumulation and oxidative stress in HepG2 cells. **(A)** T-CHO, TG, HDL-C, and LDL-C in HepG2 cells treated with HFA and 0.1 mM OA; **(B)** MDA, GSH, SOD, and CAT levels in HepG2 cells treated with HFA and 0.1 mM OA; **(C)** Oil-red O staining (20×) of HepG2 cells treated with HFA and 0.1 mM OA: **(a–e)** for control, model, simvastatin, high-dose and low-dose groups, respectively; **(f)** Statistical results of oil-red O stained areas. Data represent mean ± SD (*n* = 3). Statistically significant results were indicated as *p < 0.05, **p < 0.01, ***p < 0.001, and ****p < 0.0001 vs. control group; ^#^*p* < 0.05, ^##^*p* < 0.01, ^###^*p* < 0.001, and ^####^*p* < 0.0001 vs. model group.

According to the effect of OA on cell viability measured by CCK-8 (Supplementary Figure 2A), we chose 0.1 mM of OA to establish the HepG2 hyperlipidemic cell model. According to the effect of HFA on cell viability measured by CCK-8 (Supplementary Figure 2B), HFA of 0.25 mg/mL and 0.125 mg/mL were selected for the experiment. Compared with the control group, the TC, TG, and LDL contents of HepG2 cells were significantly increased and HDL contents were significantly decreased after adding 0.1 mM OA for 18 h (Figure 2A). Meanwhile, a significant increase in lipid droplets could be observed in the results of oil red O staining (Figure 2C). It proves that the cellular hyperlipidemia model was established successfully. In the HFA treatment group, a significant improvement of these indices could be observed; a significant decrease in the content of TC, TG, and LDL; and a significant decrease in intracellular lipid droplets and lipid accumulation. In conclusion, these results suggest that in OA-injured HepG2 cells, HFA administration reduces TC and TG content, increases HDL content, reduces the accumulation of lipid droplets in cells, and has a protective effect on HepG2 cells.

To determine the antioxidant effect of HFA on HepG2 cells, we measured the levels of MDA, GSH, SOD, and CAT in cells. MDA is cytotoxic as the end-product of lipid peroxidation by free radicals in living organisms, and it can lead to cross-linking and polymerization of macromolecules. SOD and CAT activities are important indicators to measure the ability to resist oxidation and scavenging free radicals. As shown in Figure 2B, compared with the control cells, MDA levels were significantly increased after OA injury (*P* < 0.0001). The levels of GSH, SOD and CAT were significantly decreased (*P*-values less than 0.0001, 0.001 and 0.01, respectively), indicating that the cellular antioxidant capacity was impaired and accelerated the hyperlipidemia process. HFA treatment could significantly reduce MDA levels (*p* < 0.0001) and increase the levels of GSH, SOD, and CAT (*p*-values less than 0.0001, 0.01, and 0.05, respectively). These indicators indicated the HFA has a good antioxidant capacity.

### The effect of hawthorn fruit acid on lipid levels in hyperlipidemia rats

**FIGURE 3:**
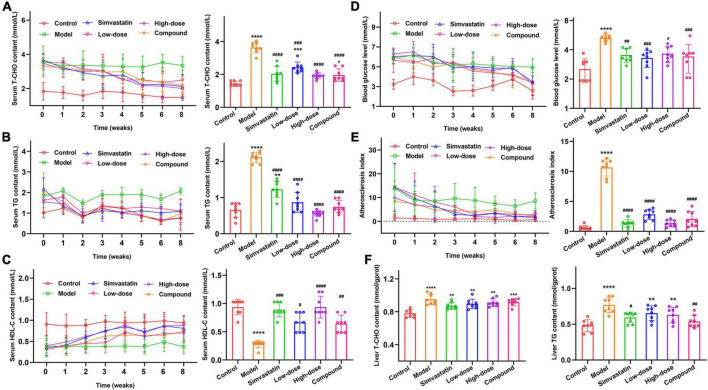
Effect of HFA on lipids in serum and liver of hyperlipidemic rats. **(A–E)** Changes in T-CHO, TG, HDL-C, GLU, and AI in rat serum during 8 weeks. **(F)** Changes in T-CHO and TG levels in rat liver homogenates. Data represent mean ± SD (*n* = 8). Statistically significant results were indicated as *p < 0.05, **p < 0.01, ***p < 0.001, and ****p < 0.0001 vs. control group; ^#^*p* < 0.05, ^##^*p* < 0.01, ^###^*p* < 0.001, and ^####^*p* < 0.0001 vs. model group.

To investigate the ability of HFA on alleviating the symptoms of hyperlipidemia rats, the hyperlipidemia rat model was established by the rats which have been fed for 8 weeks by FAD. Before HFA treatment, the plasma levels of TC and TG of hyperlipidemia rats were 3.34 ± 0.51 mmol/L and 2.07 mmol/L, respectively, which were significantly higher than those of the blank control rats (1.49 ± 0.18 mmol/L and 0.76 ± 0.31 mmol/L). This demonstrates that the model was established successfully. The hyperlipidemia rats were further treated with HFA for 8 weeks. The results showed that the serum levels of TC (Figure 3A), TG (Figure 3B), and GLU (Figure 3D) decreased gradually, and the HDL-C levels increased gradually in the HFA-treated rats over time (Figure 3C). AI values calculated by TC and HDL-C decreased simultaneously (Figure 3E), suggesting a reduced risk of cardiovascular disease. Similarly, TC and TG were reduced in the liver of HFA-treated rats compared to hyperlipidemia rats (Figure 3F). In conclusion, these results validated that HFA treatment effectively improved hyperlipidemia.

**FIGURE 4:**
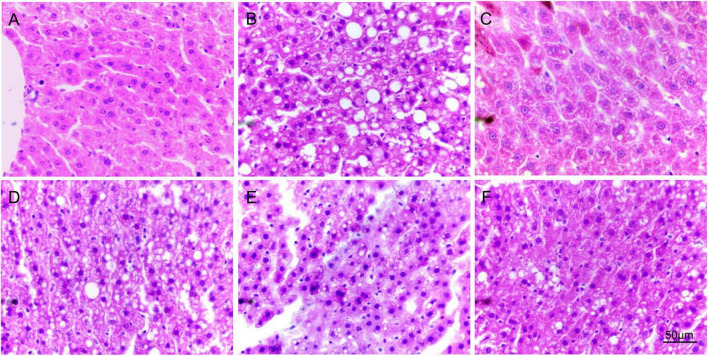
The images of H&E staining in the liver. **(A–F)** are the H&E staining pictures of liver slices in the control, model, simvastatin, low-dose, high-dose, and compound group, respectively.

To observe the fat accumulation in the rat liver, we performed a pathological examination of the rat liver. Normal rats don’t have fat accumulation in the liver. Its cells were intact and regularly arranged, and the nuclei of the cells were clear (Figure 4A). Hyperlipidemia rats (Figure 4B) showed severe changes in liver structure, such as hepatocyte necrosis, cytoplasmic vacuolization, cell degeneration, and loss of cell boundaries. Also, a large accumulation of fat in the hepatocytes was observed in the form of droplets. In the simvastatin group (Figure 4C), there was a reduction in the accumulation of intracellular fat and the cells were morphologically intact and relatively well aligned. Different degrees of fat accumulation were observed in the liver of the low and high-dose groups (Figures 4D,E). The morphology of the cells was changed and was irregularly arranged. In the high-dose group, the droplet-shaped fat particles were smaller and less numerous, and the cells were arranged more regularly. The condition of the liver in the compound group (Figure 4F) was between high and low. The results suggest that the administration of Hawthorn fruit acid extract can reduce the liver fat accumulation caused by HFD.

### Antioxidant effect of hawthorn fruit acid on hyperlipidemia rats

**FIGURE 5:**
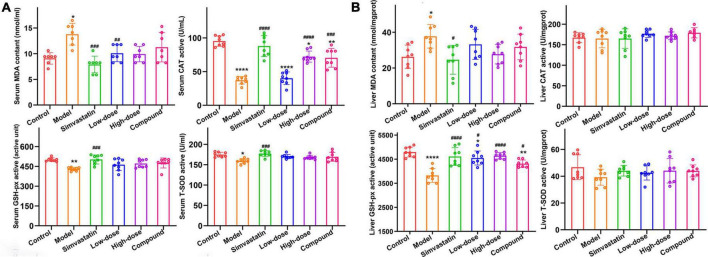
The effect of HFA on MDA and antioxidant enzymes in serum and liver of hyperlipidemia rats. **(A,B)** MDA, CAT, GSH-px and SOD levels in serum and liver, respectively. Data represent mean ± SD (*n* = 8). Statistically significant results were indicated as *p < 0.05, **p < 0.01, ***p < 0.001, and ****p < 0.0001 vs. control group; ^#^*p* < 0.05, ^##^*p* < 0.01, ^###^*p* < 0.001, and ^####^*p* < 0.0001 vs. model group.

To investigate the *in vivo* antioxidant capacity of HFA, we measured the enzymatic activities of CAT, GSH-px, SOD, the content of MDA, and T-AOC capacity in serum and liver homogenates of rats. The results showed that before HFA treatment, the serum levels of MDA, SOD, and CAT in hyperlipidemia rats were 13.78 ± 2.12 nmol/mL, 157.8 ± 10.96 U/mL, 36.91 ± 6.02 U/mL, respectively, which were significantly different from those in control rats (9.08 ± 1.12 nmol/mL, 172.7 ± 6.82 U/mL, 94.85 ± 7.81 U/mL). These phenomena were significantly improved after HFA treatment (Figure 5A and Supplementary Figure 3). A similar situation occurred in the liver (Figure 5B and Supplementary Figure 3). These results suggest that HFD leads to impaired antioxidant capacity and accelerated hyperlipidemia in rats. Administration of HFA reduces the accumulation of MDA in rats and increases the activity of antioxidant enzymes through antioxidant effects, thus reducing the damage caused by HFD in rats.

### Effect of hawthorn fruit acid on HepG2 cell protein and gene expression

**FIGURE 6:**
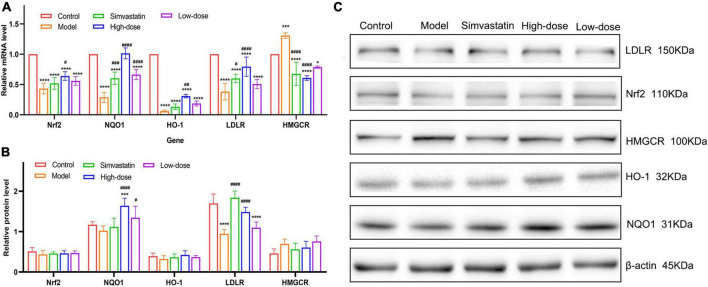
Effect of HFA on protein and gene expression in HepG2 cells. **(A)** Relative mRNA expression levels of Nrf2, NQO1, HO-1, LDLR, and HMGCR were quantified by RT-qPCR; **(B,C)** Protein expression levels of Nrf2, NQO1, HO-1, LDLR, and HMGCR. Data represent mean ± SD (*n* = 3). Statistically significant results were indicated as *p < 0.05, **p < 0.01, ***p < 0.001, and ****p < 0.0001 vs. control group; ^#^*p* < 0.05, ^##^*p* < 0.01, ^###^*p* < 0.001, and ^####^*p* < 0.0001 vs. model group.

To clarify whether the hypolipidemic effect of HFA is related to its antioxidant activity, the protein and mRNA expression of Nrf2, HO-1 and NQO1 were measured in HepG2 cells in this study. As shown in Figure 6, the relative mRNA expression of Nrf2, HO-1, and NQO1 was significantly increased in HFA-treated cells compared to OA-treated cells, along with increased protein expression. Activation of Nrf2 resulted in increased expression of HO-1 and NQO1. These results suggest that the Nrf2/HO-1 pathway is activated to protect HepG2 cells from OA-induced oxidative stress.

To verify the possible mechanism of lipid reduction by HFA administration, we measured the protein and mRNA expression of LDLR and HMGCR in HepG2 cells. LDLR is a receptor for LDL-c and contributes to the transport and clearance of cholesterol from the plasma to the cytoplasm, and HMGCR acts as a key regulatory enzyme controlling endogenous cholesterol synthesis. Both LDLR protein expression and relative mRNA expression were significantly reduced and HMGCR protein expression and mRNA expression were increased in OA-induced cells compared to control cells. HFA treatment was able to significantly alter this phenomenon. These results suggest that HFA administration may regulate cholesterol biosynthesis and cholesterol metabolism by modulating the expression of HMGCR and LDLR.

## Discussion

Hyperlipidemia is a highly prevalent risk factor for atherosclerosis and stroke, usually manifested by elevated TC and/or TG, and decreased HDL-C. In the present study, to investigate the hypolipidemic effect and mechanism of oral HFA, we first identified its main components as OxA, TA, L-MA, AA, CiA, GA, ChA, CaA, P-CA, and FA. Our results showed that HFA administration decreased TC and TG content and increased HDL-C content in HepG2 cells *in vitro*. In rats with HFD induced hyperlipidemia, after HFA treatment, the TC and TG contents gradually decreased with the duration of HFA treatment. At week 8, significant differences have been observed compared to the model group. These findings suggest that treatment with HFA administration can significantly improve the lipid levels in rats. Gallic acid, chlorogenic acid, and ferulic acid have been reported to have lipid-lowering effects (19, 20, 27); citric acid has a strong antioxidant capacity (28). The results of some studies have shown that in Sprague-Dawley rats fed with a hypercholesterolemic diet, chlorogenic acid significantly altered the increase in plasma total cholesterol and LDL (29); ferulic acid intervention significantly reduced TC, TG, and LDL-C and increased HDL-C in hyperlipidemia patients, while also significantly reducing the levels of MDA (10, 30); gallic acid alleviates hypertriglyceridemia and lipid accumulation by enhancing glycolytic and lipolytic pathways in the perirenal adipose tissue of hyperlipidemic rats (31). It is reported that the combination of weak organic acids has a synergistic function in inhibiting urinary path-ogens (32). We speculate that the good lipid-lowering activity of HFA may also be related to the synergistic effect of a variety of organic acids.

To verify the possible mechanism of oral HFA on lipid-lowering, we measured the expression of LDLR, HMGCR protein, and mRNA in HepG2 cells. In the results of this study, the expression of LDLR protein and mRNA in HepG2 cells showed an increasing trend after HFA treatment, while the expression of HMGCR protein and mRNA showed a decreasing trend. The ethanolic extract of Hawthorn was reported to inhibit the stimulatory effect of an HFD on HMGCR and p65 transcription and counteract the down-regulation of CYP7A1 and LDLR (33). Ferulic acid in the extract inhibited the expression of HMGCR, thereby decreasing TC and LDL-C (10). The results suggest that HFA administration can up-regulate LDLR expression and down-regulate HMGCR expression by regulating the metabolism of cholesterol.

Hyperlipidemia is associated with increased oxidative stress and increased lipid peroxidation (10). One study found that fat accumulation leads to an imbalance in adipocytokine production in response to oxidative stress and that selective increases in ROS produced in fat accumulation lead to elevated systemic oxidative stress (9). Many studies have shown that reducing ROS production improves insulin sensitivity, hyperlipidemia, and hepatic steatosis (34). In addition to lipid peroxidation, ROS can activate NF-κB and trigger inflammatory mediators (35). To clarify whether the hypolipidemic effect of HFA is related to its antioxidant activity, this study measured the protein and gene expression of Nrf2, HO-1, and NQO1 in HFA-treated HepG2 cells. After intracellular ROS production, the antioxidant response pathway is activated through nuclear translocation of Nrf2, which activates antioxidant response genes and induces phase II detoxification enzymes, such as HO-1 (36). HO-1 scavenges ROS, thereby preventing excessive lipid and protein oxidation, and plays an effective role in antioxidant, anti-inflammatory, and anti-apoptotic activities (37, 38). In this study, we observed that after HFA treatment of HepG2 cells both protein and mRNA expression levels in the cells were increased. Treatment with chlorogenic acid activates Nrf2 and increases the activity of antioxidant enzymes, thus antagonizing oxidative damage (37, 39, 40). Gallic acid protects against ethanol-induced gastric lesions in rats by activating the Nrf2/HO-1-associated pathway (41). Ferulic acid reduces oxidative stress, inflammation, and cell death by activating Nrf2/HO-1 signaling and PPARγ (35). These results may demonstrate that HFA administration increases the antioxidant capacity of HepG2 cells and reduces OA-induced hyperlipidemia by activating the Nrf2/HO-1 signaling pathway. Increased protein and mRNA expression of NQO1 was also observed in HepG2 cells.

In our findings, an HFD caused excessive accumulation of MDA in the rat liver. MDA is a product of lipid peroxidation. During fatty acid oxidation, the production of excessive ROS will disrupt the balance between antioxidants and oxidants in the liver (42). When ROS increase, CAT and GSH decrease, thus amplifying the effects of oxidative stress and creating a vicious cycle (43). Hyperlipidemia damages endothelial cells, triggering simultaneous macrophage invasion and lipid deposition, which are key steps in the development of atherosclerosis (44). ROS production serves as a key mediator involved in hyperlipidemia through these mechanisms: direct damage to cell membranes and nuclei and production of oxidized lipoproteins (45). Antioxidant enzymes reduce oxidant levels, thereby reducing lipid peroxidation. These enzymes known to be effective in atherosclerosis include SOD, GPx, CAT, and PON1 (45). In the study, MDA levels were significantly reduced in rats treated with HFA. The total antioxidant capacity in serum and liver of HFA-treated rats was significantly increased, and the activities of CAT, GSH-px, and SOD enzymes were also found to be on the rise. These findings suggest that oral administration of HFA can protect rats from HFD-induced liver damage and reduce the risk of atherosclerosis by reducing oxidative damage.

In epidemiological studies in Asia and Europe, hyperlipidemia is commonly associated with diabetes (46). One study showed that a short-term HFD diet in hamsters resulted in severe hepatic steatosis, glucose intolerance, and a slight increase in fasting glucose, suggesting the development of type II diabetes (47). Another study showed that, compared with participants with normal lipids, subjects with mixed hyperlipidemia had a risk of developing type II diabetes that was more than three times higher in subjects with mixed hyperlipidemia compared to participants with normal lipids (46). To confirm whether HFA administration reduces the risk of atherosclerosis and hyperglycemia, we measured GLU and AI in hyperlipidemic rats. The results showed that feeding HFD to rats for 2 weeks could lead to the increase of Glu and the change of blood lipid level, thus increasing AI. After 8 weeks of HFA treatment, GLU and AI levels in rats decreased significantly. The results of this study show that oral administration of HFA to rats can reduce blood lipid and blood glucose levels and reduce the risk of atherosclerosis caused by HFD.

## Conclusion

The results of this study showed that HFA administration was effective in preventing the progression of hyperlipidemia in a rat model, as demonstrated by its ability to attenuate the increase in serum lipid levels induced by HFD and to reduce lipid accumulation in the liver. HFA administration inhibited lipid accumulation in HepG2 cells by up-regulating LDLR and down-regulating protein and gene expression of HMGCR. HFA treatment protected hepatocytes by activating the Nrf2/HO-1 signaling pathway to protect hepatocytes. In conclusion, this study demonstrates the potential of HFA as a therapeutic agent in the clinical treatment of hyperlipidemia.

## Data availability statement

The original contributions presented in the study are included in the article/Supplementary material, further inquiries can be directed to the corresponding authors.

## Ethics statement

The animal study was reviewed and approved by the Experimental Animal Ethics Committee of Hebei Provincial Department of Science and Technology.

## Author contributions

YF conducted most of the experiments under the guidance of XD and DM. SG and TZ helped in discussions about pathology. GS helped in data analysis. PZ, YH, and SQ carried out article correction. All authors discussed the results and commented on the manuscript.
