# Supplementary material for: Hawthorn fruit acid consumption attenuates hyperlipidemia-associated oxidative damage in rats
Source: Front Nutr. 2022 Aug 3;9:936229. doi: 10.3389/fnut.2022.936229 (PMC9384962; doi:10.3389/fnut.2022.936229)
Supplement: Supplementary file 1 [file Data_Sheet_1.docx]

Supplementary Material

Hawthorn fruit acid Attenuates Hyperlipidemia-Associated Oxidative Damage in Rats

Yicheng Feng ^1,2,3^, Shan Gao^2^, Ting Zhu^3^, Guibo Sun^3^, Peisen Zhang^1^, Yichun Huang^1^, Shuang Qu^1^, Xiaomeng Du^4^*, Dehua Mou^2^*

^1^ Beijing Advanced Innovation Center for Soft Matter Science and Engineering, College of Life Science and Technology, Beijing University of Chemical Technology, Beijing 100029, China.

^2^ College of Food and Biology, Hebei University of Science and Technology, Shijiazhuang 050018, China.

^3^Institute of Medicinal Plant Development, Chinese Academy of Medical Sciences & Peking Union Medical College, Beijing 100193, China.

^4^ Beijing Obstetrics and Gynecology Hospital, Capital Medical University, Beijing Maternal and Child Health Care Hospital, Beijing 100006, China.

*** Correspondence:**Dehua Mou
dh_mou@163.com

Xiaomeng Du
duxiaomeng@mail.ccmu.edu.cn

**Supplementary Table 1. Primers used in qPCR experiments.**

| Gene | Direction | Primer sequence (5'to 3') | Number of bases(bp) |
| --- | --- | --- | --- |
| Nrf2 | forward | GACGGTATGCAACAGGACATTGAG | 24 |
|  | reverse | AACTTCTGTCAGTTTGGCTTCTGGA | 25 |
| HO-1 | forward | ACATCGACAGCCCCACCAAGTTCAA | 25 |
|  | reverse | CTGACGAAGTGACGCCATCTGTGAG | 25 |
| NQO-1 | forward | GGATTGGACCGAGCTGGAA | 19 |
|  | reverse | AATTGCAGTGAAGATGAAGGCAAC | 24 |
| GAPDH | forward | TGGACTTGCCTGTTAAATGGG | 21 |
|  | reverse | TGGTGAAGACGCCAGTGGA | 19 |
| HMGCR | forward | GTCATTCCAGCCAAGGTTGT | 20 |
|  | reverse | GGGACCACTTGCTTCCATTA | 20 |
| LDLR | forward | GAATTTGGCCAGACACAGGT | 20 |
|  | reverse | CACCGTACCCAGCTGATTTT | 20 |

**Supplementary Table 2. Composition of basic feed and high-fat diet.**

| Ingredient | Basic feed content (g/kg) | High fat feed content (g/kg) |
| --- | --- | --- |
| Corn starch | 508 | 407 |
| Casein | 242 | 242 |
| White sugar | 119 | 119 |
| lard | 50 | 150 |
| Cholesterol | 0 | 1 |
| Complex minerals | 40 | 40 |
| Compound microbiotics | 20 | 20 |
| Gelatin | 20 | 20 |
| DL-methionine | 1 | 1 |

**
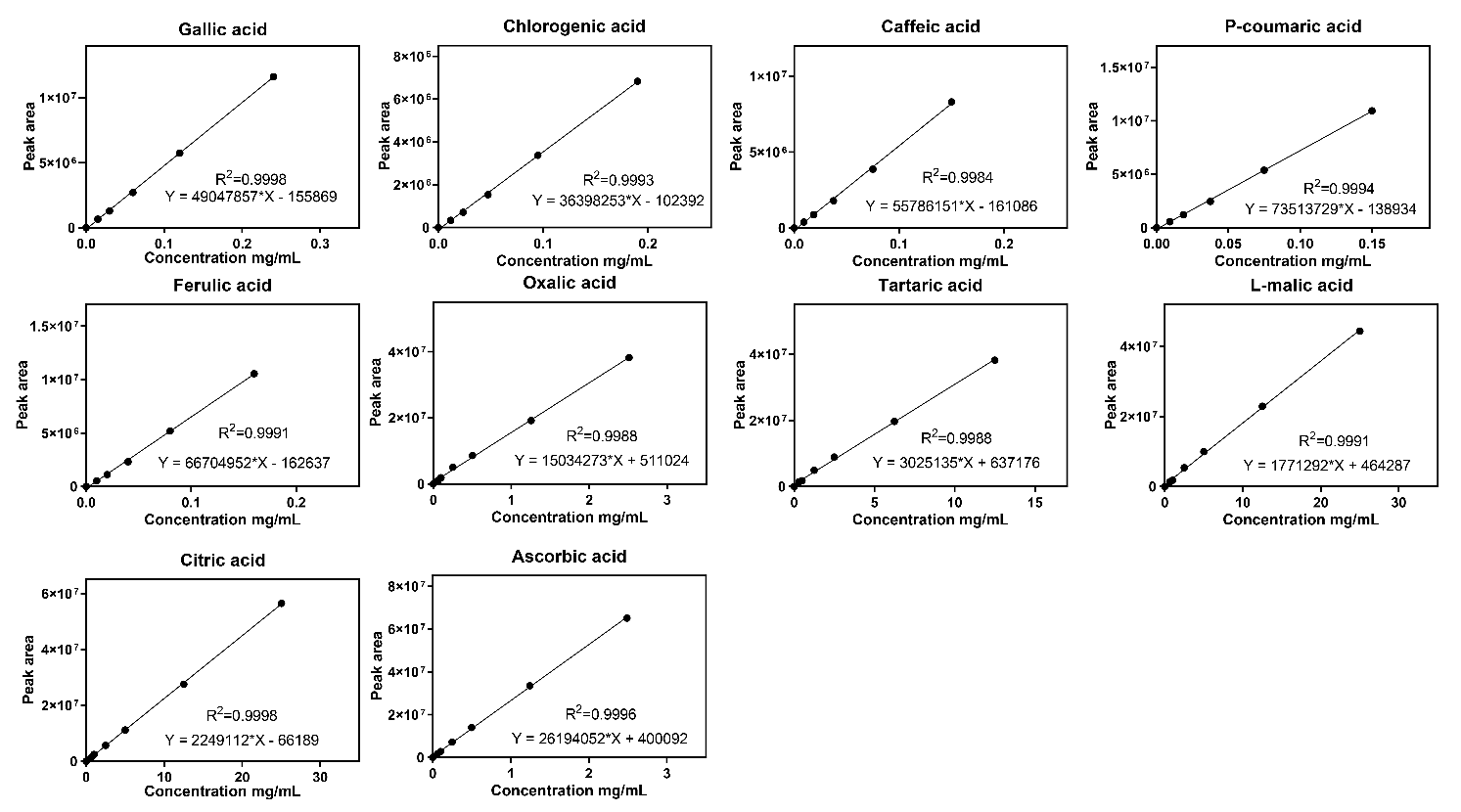
**

**Supplementary Figure 1. HPLC to establish fruit acid standard curve.**

**Supplementary Table 3. The main compounds and content of HFA**

| Serial number | Name | Content mg/g dry weight |
| --- | --- | --- |
| 1 | Oxalic acid | 0.33±0.09 |
| 2 | Tartaric acid | 11.85±0.08 |
| 3 | L-malic acid | 3.79±0.10 |
| 4 | Ascorbic acid | 1.85±0.08 |
| 5 | Citric acid | 147.67±1.29 |
| 6 | Gallic acid | 0.41±0.01 |
| 7 | Chlorogenic acid | 1.04±0.12 |
| 8 | Caffeic acid | 0.472±0.15 |
| 9 | P-coumaric acid | 0.64±0.03 |
| 10 | Ferulic acid | 0.84±0.15 |


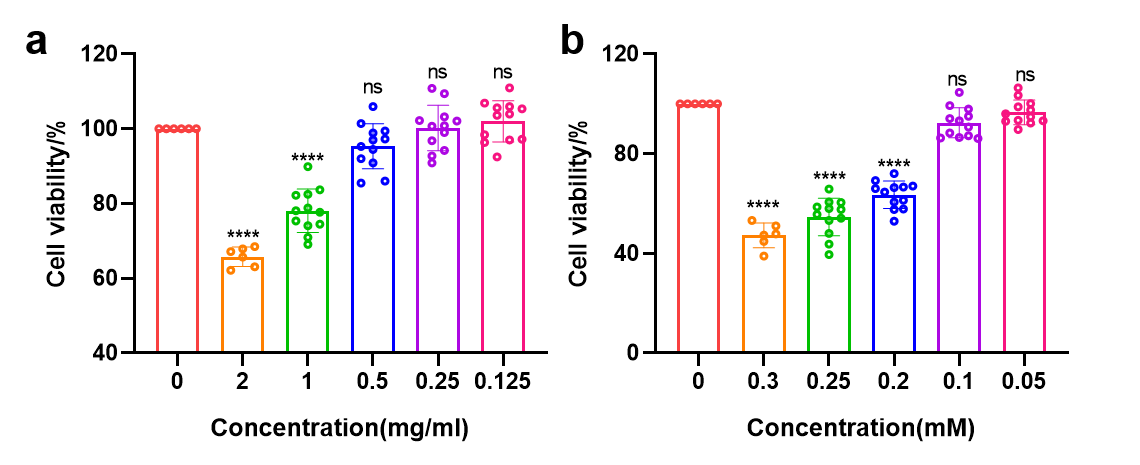


**Supplementary Figure 2. The viability of HepG2 cells in different concentrations of HFA (a) and OA (b).** The data represents the mean ± SD (n ≥ 6 in each group). *p<0.05, ** p <0.01, *** p <0.001, ****p<0.0001 vs. Control.


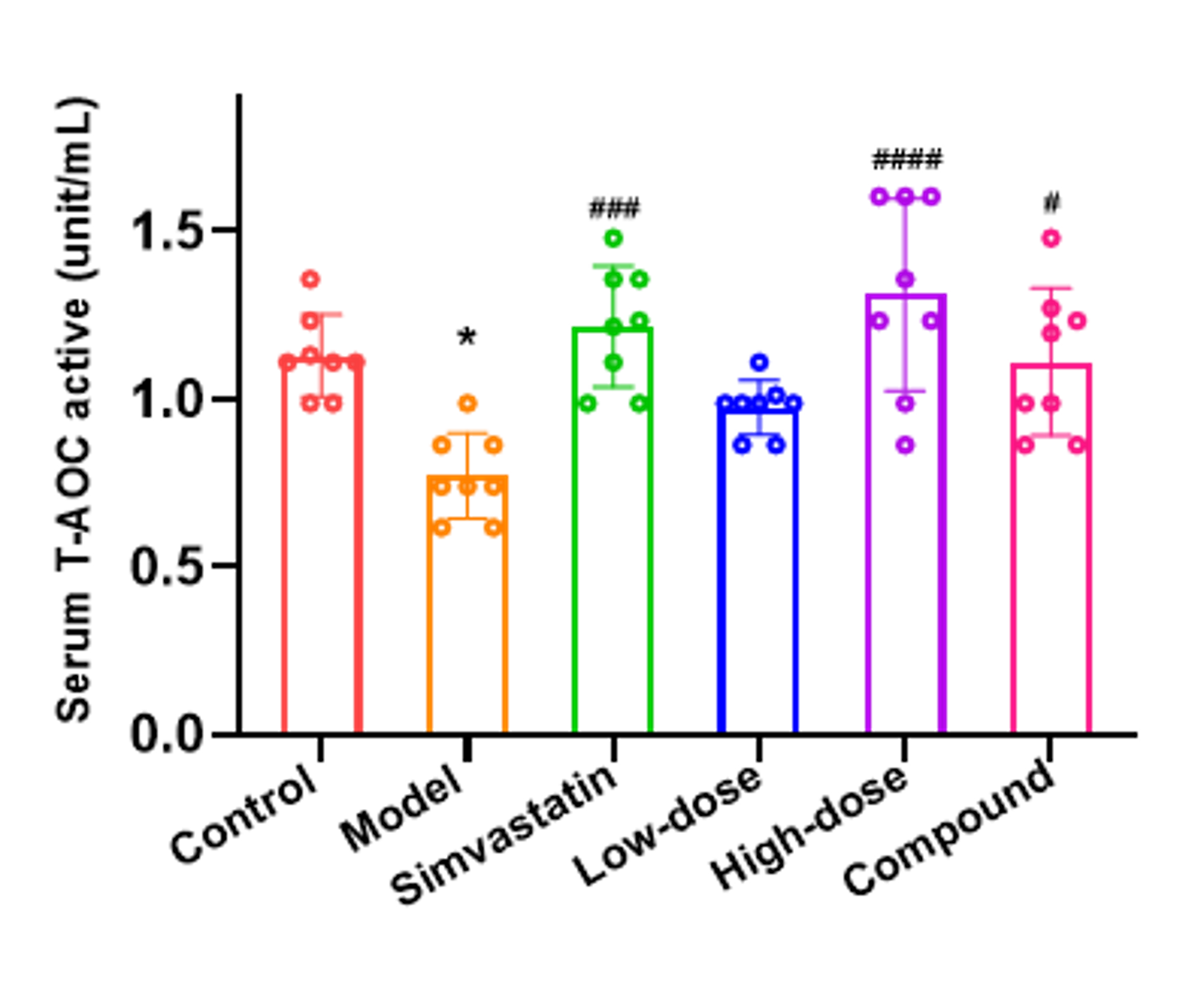

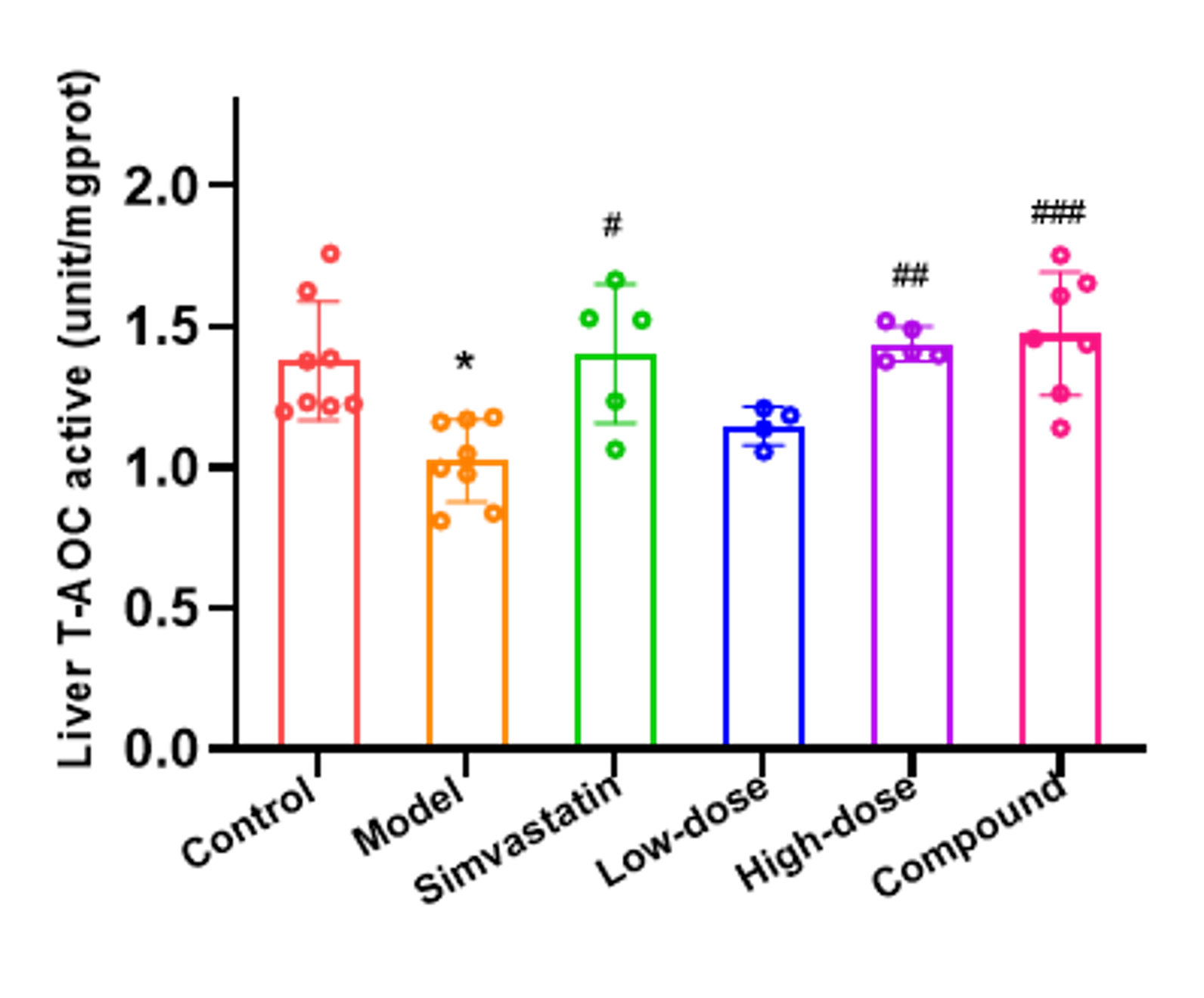


**Supplementary Figure 3. Effect of HFA on T-AOC in serum and liver of hyperlipidemic rats.** The data represents the mean ± SD (n=8).


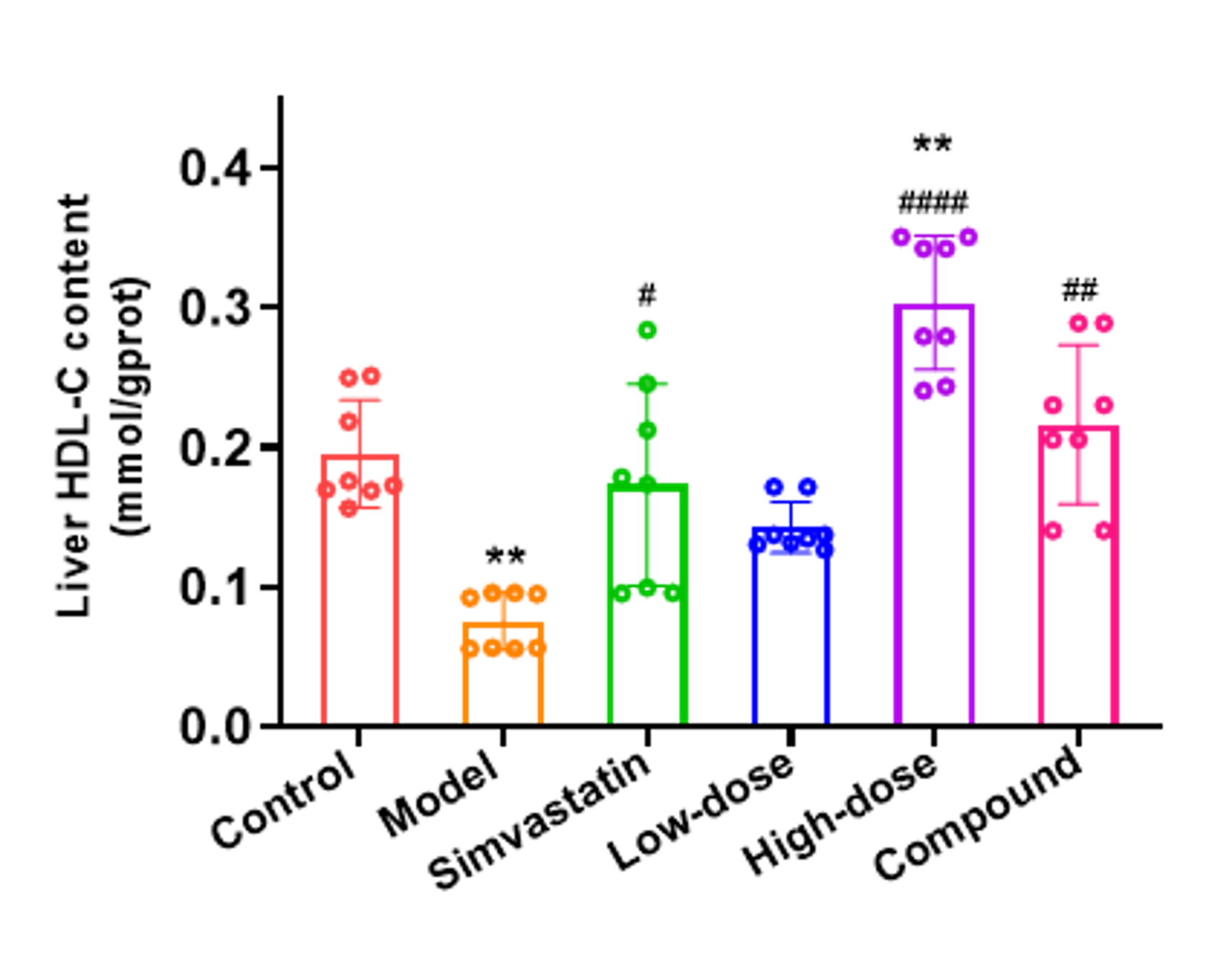


**Supplementary Figure 4. Effect of HFA on HDL-C in liver of hyperlipidemic rats.** The data represents the mean ± SD (n=8).

**
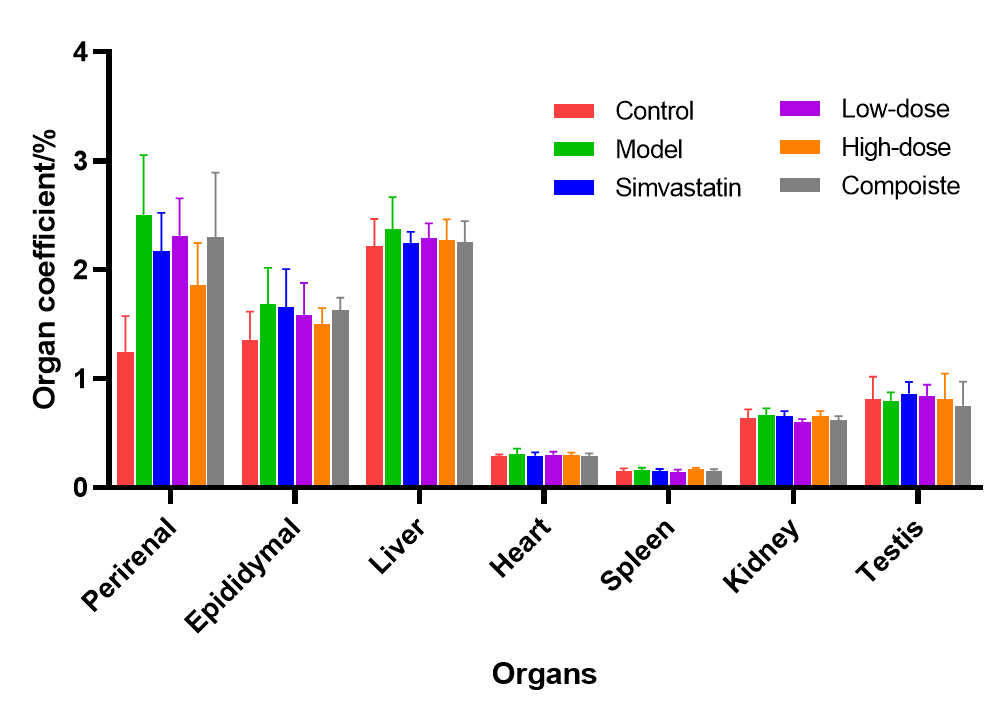
**

**Supplementary Figure 5. Animal organ coefficient.** The perirenal, epididymal, liver, heart, spleen, kidney，and testis of each group of rats were weighed, and the ratio to body weight was the organ coefficient. The data represents the mean ± SD (n=8).
